# Underlying mechanisms and cardioprotective effects of SGLT2i and GLP-1Ra: insights from cardiovascular magnetic resonance

**DOI:** 10.1186/s12933-024-02181-7

**Published:** 2024-03-11

**Authors:** Angelica Cersosimo, Nadia Salerno, Jolanda Sabatino, Alessandra Scatteia, Giandomenico Bisaccia, Salvatore De Rosa, Santo Dellegrottaglie, Chiara Bucciarelli-Ducci, Daniele Torella, Isabella Leo

**Affiliations:** 1 Department of Experimental and Clinical Medicine, Magna Graecia University, Catanzaro, Italy; 2 Advanced Cardiovascular Imaging Unit, Ospedale Medico-Chirurgico Accreditato Villa dei Fiori, Naples, Italy; 3 Department of Neuroscience, Imaging and Clinical Sciences, Institute for Advanced Biomedical Technologies “G. d’Annunzio”, University of Chieti-Pescara, Chieti, Italy; 4 Department of Medical and Surgical Sciences, Magna Graecia University, Catanzaro, Italy; 5 CMR Unit, Royal Brompton and Harefield Hospitals, Guy’s and St Thomas’ NHS Foundation Trust, London, UK; 6 School of Biomedical Engineering and Imaging Sciences, Faculty of Life Sciences and Medicine, Kings College London, London, UK

**Keywords:** Cardiovascular magnetic resonance, SGLT2i, GLP-1Ra, Diabetic cardiomyopathy

## Abstract

Originally designed as anti-hyperglycemic drugs, Glucagon-Like Peptide-1 receptor agonists (GLP-1Ra) and Sodium-glucose cotransporter-2 inhibitors (SGLT2i) have demonstrated protective cardiovascular effects, with significant impact on cardiovascular morbidity and mortality. Despite several mechanisms have been proposed, the exact pathophysiology behind these effects is not yet fully understood. Cardiovascular imaging is key for the evaluation of diabetic patients, with an established role from the identification of early subclinical changes to long-term follow up and prognostic assessment. Among the different imaging modalities, CMR may have a key-role being the gold standard for volumes and function assessment and having the unique ability to provide tissue characterization. Novel techniques are also implementing the possibility to evaluate cardiac metabolism through CMR and thereby further increasing the potential role of the modality in this context. Aim of this paper is to provide a comprehensive review of changes in CMR parameters and novel CMR techniques applied in both pre-clinical and clinical studies evaluating the effects of SGLT2i and GLP-1Ra, and their potential role in better understanding the underlying CV mechanisms of these drugs.

## Introduction

Currently, type 2 of diabetes mellitus (DM2) affects more than 500 million people [1–3].

Among the well-known systemic manifestations of type 2 diabetes mellitus (DM2), cardiovascular (CV) diseases represent the most relevant complications, accounting for the prevalent cause of morbidity and mortality [4–9]. Two classes of medications designed as novel therapeutic strategies for DM2, namely Glucagon-Like Peptide-1 receptor agonists (GLP-1Ra) and Sodium-glucose cotransporter-2 inhibitors (SGLT2i), have demonstrated to reduce CV mortality and the occurrence of heart failure (HF) in patients with DM2 [8–10]. Notably, this effect was observed with SGLT2i, regardless of the presence of DM2 [10–15]. Although the precise mechanisms underlying these cardioprotective effects remains not completely understood, several studies have proposed that they may act independently of glycemic control attributing their beneficial effects to direct as well as indirect actions on the CV system [11, 12].

**Fig. 1:**
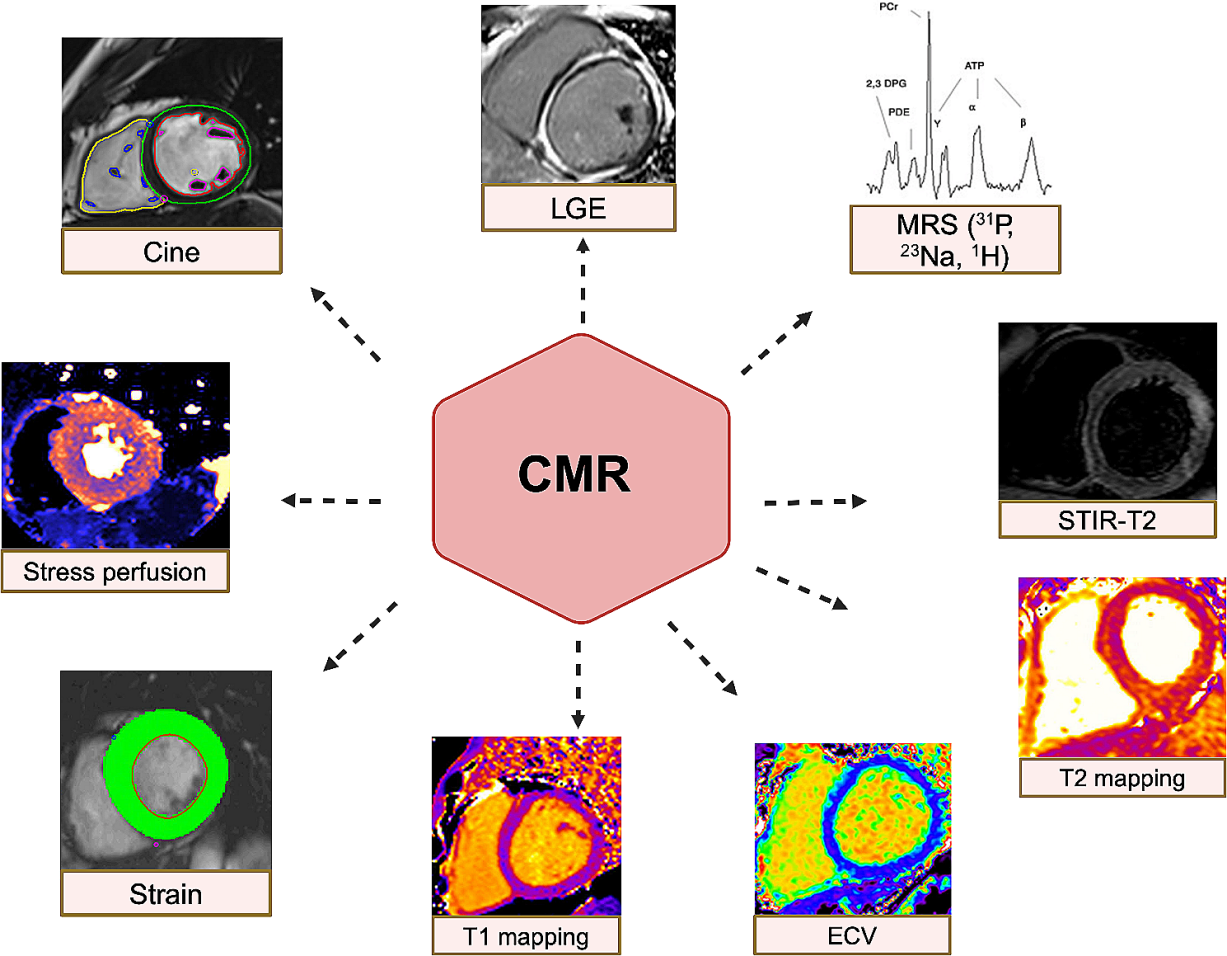
Examples of CMR sequences used to evaluate cardiovascular effects of SGLT2i or GLP-1Ra. ECV: extracellular volume; MRS: magnetic resonance spectroscopy; LGE: late gadolinium enhancement; STIR-T2: Short-TI Inversion Recovery

Cardiac remodeling, defined as changes in the cardiac geometry and/or function, often precedes the development and progression of HF and is associated with poor clinical outcomes [16–18]. The evaluation of early, subclinical, changes at CV level induced by GLP-1Ra and SGLT2i will be key to unravel their cardioprotective effects [19, 20]. Among the different imaging modalities, Cardiovascular Magnetic Resonance (CMR) may play a pivotal role in this regard, being not only the gold standard for volumetric and function assessment [21], but also providing tissue characterization with the possibility to image myocardial fibrosis/necrosis, oedema and, when applying a stress protocol, the presence of inducible myocardial ischaemia [22]. Novel CMR sequences have also been recently developed to allow a non-invasive assessment and quantification of microvascular ischaemia [23] and to image cardiac inflammation and energetics [24]. Evaluating changes in CMR parameters can therefore add meaningful piece to the puzzle describing the mechanisms of action underlying the beneficial CV effects of GLP-1Ra and SGLT2i (Fig. 1).

The aim of this article is to provide a narrative review of the existing evidence in the literature regarding the established and potential role of CMR in assessing the cardiovascular effects of GLP-1Ra and SGLT2i.

## SGLT2i effects on cardiovascular system

**Fig. 2:**
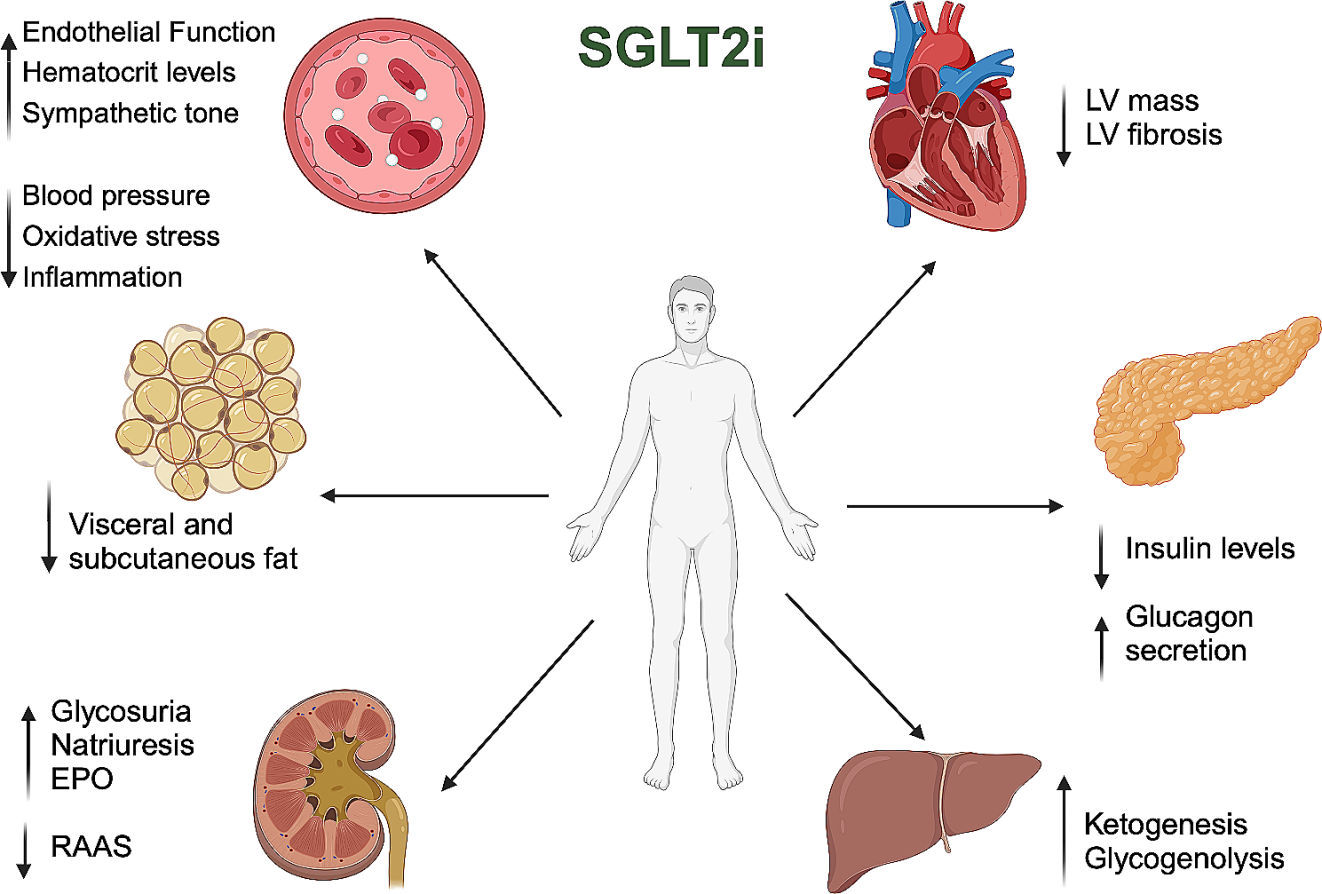
Summary of the effects of sodium-glucose cotransporter-2 inhibitors EPO: Erythropoietin. LV: left ventricular; RAAS: renin-angiotensin-aldosterone system;

Originally considered solely as hypoglycemic drugs, SGLT2i operate by reducing glucose reabsorption through the blocking of the SGLT2 receptor in the proximal renal tubule, consequently inducing glycosuria [25–27] (Fig. 2). This, in turn, reduces plasma insulin levels and promote glucagon secretion, responsible for lipolysis and lipid oxidation, with the effect of an overall reduction in visceral and subcutaneous fat and a weight loss of ~ 2-3 kg [28–34]. Additionally, the natriuretic effect of SGLT2i inhibits the renin-angiotensin-aldosterone system (RAAS), resulting in a modest reduction in both systolic and diastolic blood pressure [28, 35]. The increased diuresis, coupled with a direct promotion of erythropoiesis, contributes to the observed rise in hematocrit levels in patients receiving these medications [36–39]. However, a similar effect has been observed with other drugs that do not impact mortality [40].

The “Empagliflozin, Cardiovascular Outcomes, and Mortality in Type 2 Diabetes” (EMPAREG-OUTCOME) [41], “Canagliflozin and Cardiovascular and Renal Events in Type 2 Diabetes” (CANVAS) [42], “Dapagliflozin and Cardiovascular Outcomes in Type 2 Diabetes” (DECLARE-TIMI 58) [43] studies have provided evidence that SGLT2i reduces major renal and CV endpoints as hospitalizations and mortality due to HF in patients with DM2.

Subsequently, trials like “Dapagliflozin in Patients with Heart Failure and Reduced Ejection Fraction” (DAPA-HF) [10] and “Dapagliflozin in Heart Failure with Mildly Reduced or Preserved Ejection Fraction” (DELIVER) [13] for dapaglifozin, as well as the “Cardiovascular and Renal Outcomes with Empagliflozin in Heart Failure” (EMPEROR-Reduced) [14] the “Empagliflozin in Heart Failure with a Preserved Ejection Fraction” (EMPEROR-Preserved) [15] trials for empaglifozin, have demonstrated a reduction in CV events regardless of LV ejection fraction (EF) and the presence of DM2.

Beneficial effects on reduced HF hospitalization and mortality have been described also in patients with history of prior myocardial infarction (MI), although safety and efficacy of these therapies early after acute MI remain uncertain. The Emmy trial [44] in fact demonstrated significant reduction of NT-proBNP with early initiation of empagliflozin after MI, while treatment with dapaglifozin in the DAPA-MI had only limited impact on CV outcomes including HF hospitalization and CV death, with a benefit observed only in terms of cardiometabolic outcomes [45]. Ongoing trials will provide more insights about the role of SGLT2i in this context [46]. Promising results in terms of MI and stroke risk reduction have been instead demonstrated with the SGLT1/2 inhibitor sotaglifozin [47, 48], with a benefit similar to what observed with GLP1Ras but with the advantage of an additional proved reduction in HF-related hospitalization. Reduction in visceral obesity, increased atherosclerotic plaque stability, and gut microbiome modulation are all potential mechanisms that may contribute to this protective effect [49–51]. Consequently, these drugs are now recommended as a cornerstone of HF treatment by the European Society of Cardiology (ESC) guidelines [10–15]. While the improved glycemic control, lowered blood pressure levels and observed weight reduction after SGLT2i treatment all contribute to improved clinical outcomes, none of these factors can fully explain the overall beneficial effect on the CV system. The improvement of endothelial function and arterial wall stiffness, attributed to increased vasodilation and nitric oxide production, as well as the inhibition of oxidative stress and inflammation [52–55] have been proposed as additional potential mechanisms and described in both animal and clinical models after SGLT2i treatment [56–58]. Modulation of endothelial dysfunction may be also implicated in the amelioration of renal function observed even in the absence of diabetes [59]. Furthermore, SGLT2i reduce circulating catecholamine levels [60] and impact myocardial remodeling and fibrosis, through modulation of several chemokine pathways (IL-6, TNF-α, monocyte chemoattractant protein-1), calcium homeostasis [61, 62], authophagy [60, 63–68] and RAAS inhibition [69] in pre-clinical models. All these proposed mechanisms will be discussed in detail in the following paragraphs.

## Cardioprotective effects of SGLT2i in preclinical models

The cardioprotective SGLT2i effects have been investigated in animal models with and without DM2 [69–71]. SGLT2i proved to reduce myocardial hypertrophy, fibrosis and cardiomyocyte apoptosis and, in HF models, to improve systolic function, cardiac dilatation and reduce both atrial and ventricular fibrosis [72, 73].

These results were confirmed in non-diabetic, doxorubicin-treated mice where the treatment with doxorubicin prevented the deterioration of early LV function parameters, such as geometrical deformation indices [74]. The study also showed for the first time expression of SGLT-1 receptors in the heart, opening the way for clinical testing of SGLT-1/2 antagonists, such as sotagliflozin with favorable results both in diabetic [48] and non-diabetic HF patients [47].

To further explore the mechanisms behind the effects of SGLT2i on cardiac remodeling, several studies have utilized the information arising from CMR imaging.

For instance, the effects of a two-month course of empaglifozin on diastolic function were evaluated in a porcine model of nondiabetic HF induced by occlusion of proximal left anterior descending artery [75]. Semiautomatically generated LV filling profiles were used to derive values of peak filling rate and first filling volume to estimate the amount of ventricle filled during either LV active relaxation or suction [76]. Both parameters were found to be higher in SGLT2i-treated animals, reflecting a beneficial effect on diastolic function in this group. Additionally, the reduction in left atrial volume compared to controls suggested a decrease in left atrial pressure after SGLT2i treatment. We know from previous work that two main mechanisms have been recognized in the development of diastolic disfunction: increased interstitial fibrosis and augmented cardiomyocyte stiffness [77]. Interestingly, empaglifozin-treated pigs had reduced intramyocardial fibrosis demonstrated by lower collagen deposition and decreased extracellular volume measured at T1 mapping and ECV analysis [75]. Empaglifozin was also able to improve nitric oxide signaling and impact titin phosphorylation with beneficial effects on cardiomyocyte stiffness [75].

Other potential mechanisms with a proved role in cardiac remodeling are disturbances in ionic homeostasis [78]; elevated myocardial intracellular sodium ([Na+]i) has been found in models of HF and diabetic cardiomyopathy (DC), and linked to detrimental effects on mitochondrial function and myocardial energetics [79, 80]. The ([Na+]i) overload activates in fact the Na^+^/Ca^2+^ exchanger, with increased efflux of calcium from the mitochondria to the cytosol and increased calcium influx from the extracellular environment. The result is an overall rise in intracellular calcium, disrupted calcium gradients, and subsequent disturbances in oxidative phosphorylation and ATP levels [81]. Moreover, as most cardiac contractile proteins are calcium-sensitive, calcium plays a pivotal role in maintaining efficient excitation-contraction processes [82]. Disturbances of calcium homeostasis may therefore explain, at least in part, the impairment of contractile function observed in Diabetic Cardiomyopathy (DC) [24, 77–80, 83].

Accordingly, magnetic resonance spectroscopy (MRS) is a new imaging technique providing in vivo metabolic information of the examined tissue [24, 69, 84]. By exploiting the unique signal generated by different nuclei, MRS enables the detection of several metabolites and offers non-invasive assessment of myocardial energetics. For instance, phosphorus-31 Nuclear MRS (^31^P-MRS) can track myocardial PCr/ATP ratio (a marker of the myocardial energetic state), often compromised in DM2 patients [24, 85, 86]. Using both ^31^P and ^23^Na MRS, Croteau et al. [79] demonstrated decreased PCr/ATP ratio and elevated ([Na+]i) in a mice model of DC. A one-month treatment with ertugliflozin corrected the ([Na+]i) increase, improved the PCr/ATP ratio, and reversed myocardial hypertrophy, diastolic and systolic dysfunction [79, 80].

Ongoing research employing a novel imaging technique, manganese-enhanced magnetic resonance imaging, may soon provide insights into the effects of SGLT2i on the homeostasis of another ion, calcium (NCT04591639). The technique exploits the ability of manganese, a calcium analogue, to significantly impact the T1 relaxation time, allowing for the identification of myocardial areas with normal calcium handling.

Chronic glucose overload and ectopic lipid accumulation have both been observed in DM2 and linked to HF development [82]. However, their exact contribution to myocardial dysfunction remains unclear. Joubert et al [87] sought to address this question by using a lipodystrophic mouse model, devoid of lipotoxic features, to demonstrate that glucotoxicity itself can trigger cardiomyopathic changes including LV hypertrophy and diastolic dysfunction. CMR images showed increased wall thickness, mildly reduced EF and impaired longitudinal strain in these mice, alterations that were corrected by subsequent administration of glucose-lowering drugs. Interestingly, in this model, the effects of dapagliflozin on cardiac remodeling were superior to those induced by pioglitazone. Despite both drugs counteract glucotoxicity and reduce the amount of advanced glycation end-products, these results suggest that other metabolic pathways may be implied in the benefits observed with SGLT2i. One postulated hypothesis revolves around a shift in cardiac metabolism from fatty acid and glucose oxidation (the primary sources of fuel under physiological conditions but impaired in DM2 and HF) towards the more efficient utilization of ketone bodies [84]. At this regard, Hyperpolarized [3–^13^ C]acetoacetate, a novel ketone probe applied to MRS to track the conversion of [3-^13^C]acetoacetate into its metabolic products, was used to test the effects of empaglifozin in diabetic rats with HF. Despite an increase in the overall amount of circulating ketone bodies, their use at the cardiac level after empagliflozin administration remained surprisingly stable. Nevertheless, the drug once again confirmed a significant impact on afterload (reduced EDV and stroke volume at CMR analysis) [88]. However, another study using ^31^P-MRS to measure cardiac PCr/ATP levels as a marker of myocardial energetics [89], demonstrated a 45% increase in cardiac PCr/ATP in diabetic mice treated with a single dose of empaglifozin, correlating with the increase of circulating ketones but not with plasma glucose levels [85]. The results underscore the role that changes in myocardial energetics towards more efficient pathways may have in the cardioprotective effects of SGLT2i.

As previously mentioned, CMR is an invaluable imaging modality due to its ability to characterize tissue. In the ischemic setting this unique property allows for the identification of myocardial oedema in T2-weighted (T2w) sequences (area at risk) and myocardial scar (infarcted area) in late gadolinium enhancement (LGE) sequences. One of the potential protective mechanisms implicated in the reduction of cardiovascular events observed after SGLT2i treatment may involve the impact of these drugs on reducing post-ischemic damage. Pre-treatment with empaglifozin for one week in mice with acute myocardial infarction (MI) resulted in a significantly larger myocardial salvage area (identified by the difference between the area at risk -hyperintense in T2w- and the infarcted area measured at LGE), smaller infarct size, and overall improved cardiac function [90].

## Cardioprotective effects of SGLT2i in clinical models

One of the key findings in CMR studies involving patients treated with SGLT2i is the beneficial effect on cardiac remodeling [32, 91]. A recently published metanalysis [92] of 9 randomized clinical trials and 1385 patients reported that SGLT2i treatment significantly reduced both LV end-diastolic volume (LVEDV) and LV end-systolic volume (LVESV) as well as LVM and LVM index. Patients treated with SGLT2i also had a significant improvement on LVEF, irrespective of the time to follow-up used or of the HF phenotype. In DM2 patients, the effect on LVMi is also independent from the diabetes duration [93]. SGLT2i had instead no effect on LVM and LVMi in a cohort of non-diabetic patients, with LVH but no HF [94]. If these changes result from primarily alterations in cardiomyocyte size, extracellular volume, or a combination of both is still matter of debate [95–98]. The use of CMR imaging that with T1 mapping analysis has the potential to estimate ECV, may be the appropriate tool to non-invasively provide answers to this question. In a pre-specified analysis of the EMPA-HEART [99] both ECV and indexed ECV were significantly reduced in diabetic patients treated with empaglifozin compared to placebo. Intracellular volume (ICV), calculated as (1-ECV) x (LVMi/1.05), did not differ significantly between the two groups [99]. This 1.4%, reduction in ECV in a relatively short time frame (6 months) is particularly relevant when read in the light of the data published by Wong et al [100], where a 3% increase in ECV in diabetic patients was associated with a 52% increase in the risk of death or HF hospitalization. The reduction of ECV was confirmed by another study in non-diabetic patients, where the authors also demonstrated a reduction in cardiomyocyte volume after empaglifozin treatment [32, 101]. Ongoing trials (NCT03782259, NCT04490681) will provide further evidence about the impact of SGLT2i on ECV.

Preclinical models have highlighted the role that a shift towards more efficient energetic pathway can have in the benefit observed with SGLT2i therapy. However, similar studies using ^31^P-MRS to measure cardiac PCr/ATP levels at rest and during dobutamine stress failed to prove significant changes in cardiac energetics in both HFrEF and HFpEF [101]. Interestingly, what is significantly reduced after SGLT2i treatment is the amount of epicardial and subcutaneous adipose tissue, associated with a concomitant reduction in circulating inflammatory biomarkers [32]. Epicardial adipose tissue (EAT) serves as a lipid storage and its reduction may represent an indirect proof of the switch of myocardial fuel triggered by SGLT2i [102]. Notably, excess or abnormalities of EAT are linked to increased CV risk [103]. The EMPACEF study [104] however did not confirm the impact of empaglifozin on myocardial or epicardial fat. These conflicting results may be explained by the shorter treatment received in the EMPACEF study (12 weeks) [104], compared to the 6 months used in the EMPA-TROPISM study [32]. The reduction in aortic stiffness demonstrated after SGLT2i, with consequent reduced afterload and improved cardiac efficiency, may represent an additional mechanism involved in the overall beneficial effects in terms of CV risk [32]. Despite the undeniable benefits demonstrated in HF patients, there are mixed data regarding the effect of SGLT2i on LVEF [32, 65, 105–107]. The reasons behind these conflicting results may be the heterogeneity of patients’ selection in published studies, often with small sample size used, no stratification by EF subgroup, NYHA class distribution and degree of LV dilatation. Further studies are certainly needed to better highlight the impact of these features on efficacy of SGLT2i in the clinical setting.

Table 1 summarizes the major findings of the studies discussed in this section.

**Table 1:** Studies assessing clinical cardioprotective effects of SGLT2i by Cardiac Magnetic Resonance

Study	HF	Diabetes	SGLT2i	Duration of Therapy		Imaging Findings	
*Santos-Gallego et al.* [32]	HFrEF	No	Empaglifozin	6 months	Improvement of LV volumes, LV mass, LV systolic function, functional capacity		
*Brown et al.* [91]	No	Yes	Dapaglifozin	12 months	LVM Reduction		
*Connelly et al.* [94]	No	No	Empaglifozin	6 months	No change in LV volumes and function		
*Mason et al.* [95]	No	Yes	Empaglifozin	6 months	LVMi and ECV reduction		
*Cohen et al.* [96]	No	Yes	Empaglifozin	6 months	Reduced EDV; No changes in ESV, EF, LVM or markers of cardiac fibrosis		
*Hsu et al.* [97]	No	Yes	Empaglifozin	6 months	No improvement in LV function, structure, adiposity, and diffuse fibrosis		
*Oldgren et al.* [98]	No	Yes	Dapaglifozin	6 weeks	Reduced LA volume. Decreased Peak global radial strain. No changes in peak global longitudinal and circumferential strains. Unchanged cardiac fatty acid uptake		
*Verma et al.* [99]	No	Yes	Empaglifozin	6 months	LVMi Reduction		
*Hundertmark et al.* [101]	HFrEF/HFpEF	No	Empaglifozin	12 weeks	No improvement in cardiac energetics (PCr/ATP) at rest and during stress		
*Gaborit et al.* [104]	No	Yes	Empaglifozin	12 weeks	No change in LVM, LVEF, epicardial fat, diastolic function.		
*Lee et al.* [106]	HFrEF	Yes	Empaglifozin	36 weeks	LV volumes reduction		
*Singh et al.* [107]	HFrEF/HFpEF	Yes	Dapaglifozin	12 months	No effect on LV remodeling		

***Legend to*** Table 1: CMR: cardiac magnetic resonance; EDV: end diastolic volume; ESV: End systolic volume; ECV: extracellular volume; HFpEF: heart failure with preserved ejection fraction; HFrEF: heart failure with reduced ejection fraction; LA: left atrial; LGE: late gadolinium enhancement; LVEF: left ventricle ejection fraction; LVM left ventricular mass; LVMi: left ventricular mass index; PCr/ATP: phosphocreatine/ATP ratio

## GLP-1Ra effects on cardiovascular system

In 2005 GLP-1Ra have been approved to treat DM2 [108]. Although with different structure, duration of action, mode of administration and clinical effectiveness, these drugs overall act similarly by inducing a glucose-dependent insulin release and glucagon suppression [108–113].

In addition, they slow gastric emptying and, by their influence on central nervous system, reduce body weight [114]. GLP-1 receptors have been found in both the glomerulus and renal tubule and use of GLP-1Ra has been associated with increased natriuresis, diuresis, reduced albuminuria and suppression of the RAAS [115–119].

Beyond the metabolic effect, a significant reduction of major adverse CV events (MACE) was observed in patients treated with some of these drugs estimated at 14% when using as outcome a compositum of CV death, nonfatal MI and nonfatal stroke [115].

The underlying mechanisms are still object of current research. Surely multifactorial, they encompass physiological changes of multiple organs involved in central metabolism, systemic regulation of energy expenditure and inflammation and multiple hemodynamic factors, including modulation of blood pressure, heart rate, myocardial geometry and function, endothelial function, vascular tone and regulation of blood volumes [120].

However, not all the GLP-1 Ra are equal when looking at cardioprotection. Lixisenatide, a short acting GLP1Ra failed to demonstrate CV benefits, while liraglutide, semaglutide, dulaglutide and efpeglenatide demonstrated to lower CV events [116]. Reduced mortality was also noted with liraglutide, semaglutide and exenatide [117]. Protective effects in HF patients are controversial, with limited and non-homogenous evidence among the different molecules. The results of the FIGHT and LIVE trial in fact failed to demonstrate a protective effect of liraglutide in patients with both acute and chronic HF, respectively [121, 122]. Moreover, in a post-hoc analysis of the REWIND trial, dulaglutide administration did not reduce HF-related events [123]. Nevertheless, treatment with semaglutide demonstrated to improve HF related symptoms in non-diabetic patients with HFpEF, and a recent meta-analysis encompassing eight trials and 60,080 patients demonstrated an overall reduction of HF-related hospital admission by 11% [115, 124]. The exact reasons of these heterogeneous results are still unknown although may be partially explained by a dose-response effect, with greater CV protection being detectable only when using higher doses of the drug [125].

**Fig. 3:**
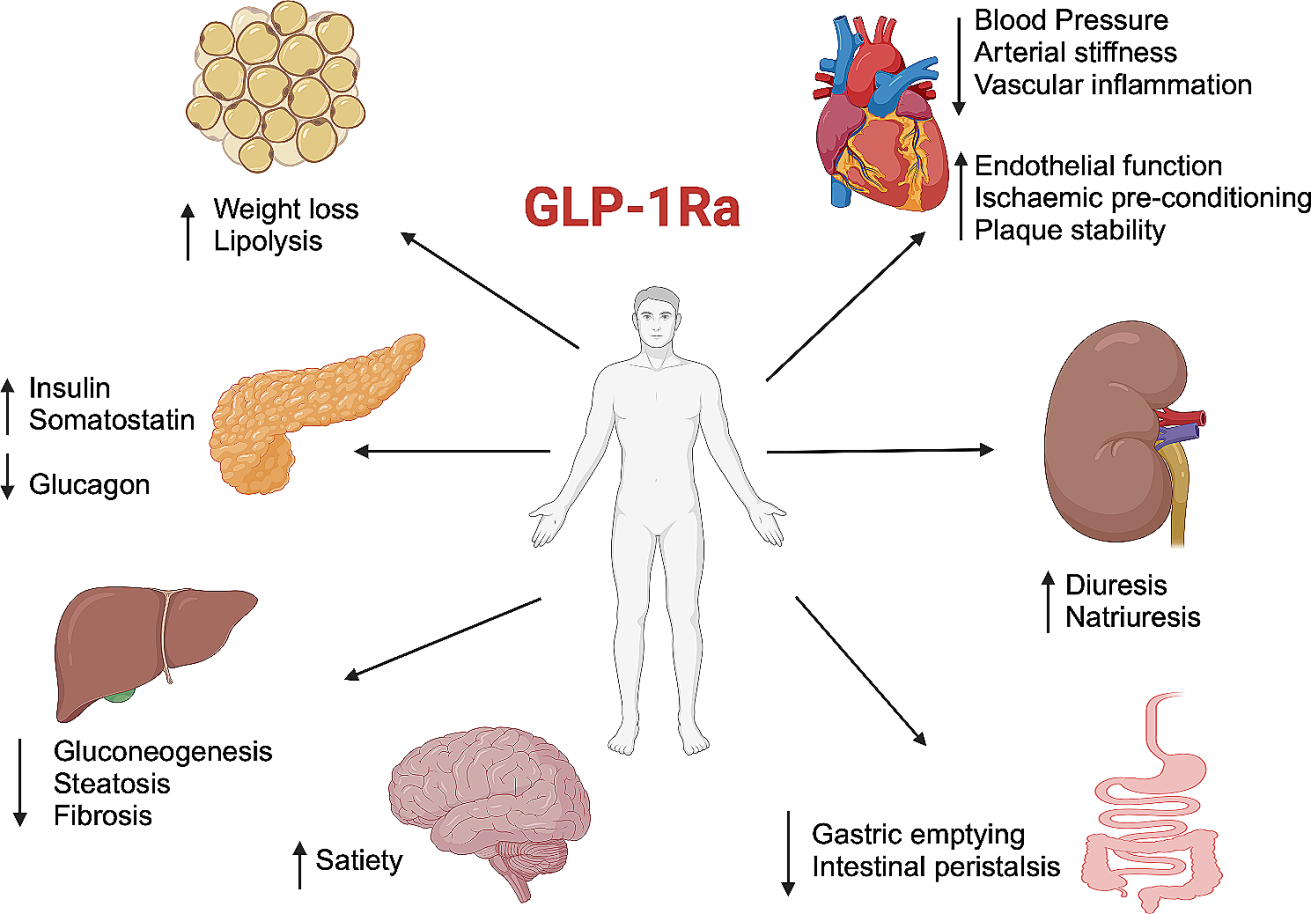
Summary of the effects of Glucagon-Like Peptide-1 receptor agonists

Following this evidence, the 2019 European Society of Cardiology (ESC) Guidelines on diabetes, pre-diabetes, and cardiovascular diseases, advise the use of GLP1-RAs in class I DM2 patients at high CV risk to reduce CV [126]. The indication was later confirmed by the 2021 ESC Guidelines on cardiovascular disease prevention (i.e. class I indication for GLP1-R in patients with DM2 and atherosclerotic cardiovascular disease to reduce CV and cardiorenal outcomes) [127]. Data published so far suggests that the overall benefit observed is mediated by a decrease of atherosclerosis-related event [128–130]. Again, the mechanism behind these effects seems to be various and not yet fully understood (Fig. 3). They have a beneficial effect on systolic blood pressure, although the reduction is only modest (2–6 mmHg) and insufficient to explain by itself the overall effects on CV mortality [119]. GLP-1Ra also lower total and LDL cholesterol and triglycerides [125, 131]. However, it seems the reduction in atherosclerosis development and progression with plaque stabilization and reduced inflammation the most critical factor in terms of CV risk reduction [129, 130].

## Cardioprotective effects of GLP-1Ra in preclinical models

One of the postulated hypothesis about the protective cardiovascular effects of GLP-1Ra look at the potential detrimental effects of lipotoxicity on cardiac function. It is in fact proved that ectopic fat accumulation and the subsequent imbalance of fatty metabolism is linked with organ damage [119, 132]. In addition, ectopic cardiac fat build-up is strictly related to the development of cardiac dysfunction [131]. The dysregulation of β-oxidation with an excess of availability of its metabolic by-products, is known to cause an increase in reactive oxygen species, contributing to oxidative stress [133]. This pro-inflammatory milieu eventually impacts calcium homeostasis with a direct effect on cardiac function. In addition, cardiac steatosis increases the amount of intramyocardial collagen resulting in reduced relaxation and diastolic dysfunction [134]. However, the exact role of lipotoxicity in the development and progression of DC remain not completely understood, underscoring the need of advanced techniques able to fill this gap in evidence. CMR imaging provides a promising no-invasive approach. Beyond the accurate heart function assessment, proton magnetic resonance spectroscopy (^1^H-MRS) is effective in detecting triglycerides within the myocardium, with good accuracy when compared to biochemical assays [135, 136]. In addition, microvascular disease is a well-known hallmark of DC that can impact the CV prognosis of patients affected by DM2 [84, 137, 138]. CMR is also helpful to non-invasively assess myocardial blood flow and microvascular ischemia [23]. In detail, arterial spin labeling (ASL) CMR has been used in animal models to assess myocardial blood flow without the use of any contrast agent [139]. Applying this multiparametric CMR protocol (^1^H-MRS and ASL) Abdesselam et al [140] demonstrated that cardiac abnormalities induced in mice after a 4-weeks course of a high-fat high-sucrose diet (i.e. cardiac hypertrophy, lower cardiac output and decrease myocardial blood flow), were reversed by a 14-day course of the GLP1-Ra Exendin-4. The drug reduced both the myocardial triglyceride content and the myocardial wall thickness [140]. At the same time, GLP1-Ra treatment was able to restore cardiac index and myocardial perfusion [140]. It has been also postulated that GLP1-Ra may exert CV beneficial effects by impacting post-MI cardiac remodeling and ischemia-reperfusion (IR) injury [141, 142]. Ischemic remodeling involves complex interactions at the cellular and molecular levels that lead to oxidative stress, inflammation, and drastic changes in pH and calcium levels, all contributing to cardiomyocyte death and excessive fibrosis [143–146]. Exenatide was found to enhance antioxidant enzyme activity, reduce oxidative stress, and decrease cell death in pigs shortly after IR injury, with similar findings in rats [141–147]. This protection against IR injury seems however to be lost in more severe models, when prolonged provoked ischemia results in irreversible damage [148, 149]. During induced ischemia in experimental models, GLP-1 was in fact able to prompt an increase in anaerobic glycolysis in the ischemic regions, to counteract the lack of oxygen supply as demonstrated using a 1-^13^C glucose clamp combined with MRS-based isotope analysis [150]. In areas with no ischemia and better oxygenation, a metabolic shift toward carbohydrate oxidation was also observed; the more energy-efficient process may help in sustaining cardiac muscle contraction in these specific circumstances.

## Cardioprotective effects of GLP-1Ra in clinical models

There are currently few and conflicting CMR data about cardioprotective effects in vivo of GLP-1Ra.

Exenatide (alone or combined with a remote ischemic conditioning approach) failed to demonstrate a beneficial effect in terms of infarct size measured by LGE, myocardial salvage index, transmurality index, LVEF and MVO volume in patients with ST-segment elevation MI receiving primary percutaneous coronary intervention (pPCI) [151]. However, in another study enrolling 172 STEMI-patients using as endpoint CMR salvage index derived from myocardial area at risk in the acute phase, and infarct size by LGE at follow-up (90 ± 21 days after pPCI) exenatide treatment resulted in a significantly larger salvage index and a smaller infarct size (when related to the myocardial area at risk), despite no differences in LVEF or significant changes in the absolute infarct size [147]. Exenatide treatment did not changed significantly LVEF, myocardial perfusion or oxidative metabolism in T2DM patients with LV systolic dysfunction, having an overall similar effect of glargine insulin [152]. Patients with acute MI treated with liraglutide demonstrated instead smaller LVMi suggesting a role in reverse remodeling [153]. A significant effect on diastolic function was instead noted on a study using liraglutide [154] that demonstrated at a CMR analysis using a 4D flow dataset with retrospective valve tracking, improved early (E) and late (A) trans-mitral peak flow rate, E/A ratio values, along with improved early deceleration peak, early peak mitral annular septal tissue velocity (Ea) and estimated LV filling pressure (E/Ea). The LVEF values were slightly reduced, although remaining within normal range.

Finally, one of the postulated hypotheses was that, given the observed reduction in body weight, GLP1-RA could induce concomitant reduction in epicardial adipose tissue (EAT). This was proven in a cohort of T2DM obese patients, where at CMR analysis EAT thickness was significantly reduced by both exenatide [155] and liraglutide [116, 156] treatment. This result was not confirmed in another study evaluating the effects of liraglutide versus placebo on DM2 patients that showed no significant change in EAT or in myocardial triacylglycerol content (a marker of myocardial steatosis) at proton MR spectroscopy [154]. Further larger studies are therefore needed to assess the impact of GLPR1a on EAT and their impact on LV function.

Table 2 summarizes the major findings of the studies discussed in this section.

**Table 2:** Studies assessing clinical cardioprotective effects of GLP1Ra by Cardiac Magnetic Resonance

Study	Heart Failure	Diabetes	GLP-1Ra	Duration of therapy	Results		
*Del Blanco et al.* [151]	No	No	Exenatide	Premedication before revascularization		No changes in infarct size measured by LGE, myocardial salvage index, transmurality index, LVEF and MVO	
*Lønborg et al.* [157]	No	No	Exenatide	15 min before intervention-6 h after the procedure		Increased myocardial salvage index and reduced infarcted size	
*Chen et al.* [152]	HFrEF	Yes	Exenatide	26 weeks		No improvement in LV function, structure, adiposity, and diffuse fibrosis	
*Nozue et al.* [153]	No	No	Liraglutide	6 months		Prevention of the progression of LV remodeling	
*Bizino et al.* [158]	No	Yes	Liraglutide	26 weeks		Reduction of diastolic and systolic function	
*Dutour et al.* [155]	No	Yes	Exenatide	26 weeks		EAT reduction	
*Zhao et al.* [116]	No	Yes	Liraglutide	3 months		EAT reduction	
*Bizino et al.* [154]	No	Yes	Liraglutide	26 weeks		No changes in EAT and myocardial triacylglycerol content	

**Legend to** Table 2: CMR: cardiac magnetic resonance; EAT: epicardial adipose tissue; LV: left ventricular; MVO: microvascular obstruction

## Conclusion

The exact mechanisms underlying the beneficial CV effects of SGLT2i and GLP1-RA are not yet completely understood. Several hypotheses have been formulated and tested in preclinical and clinical studies with the aid of CMR imaging, increasingly used in this setting due to its unique ability to provide accurate volumetric and function assessment complemented with tissue characterization. Beyond visualization and quantification of myocardial fibrosis and oedema, the most recent CMR techniques developed to assess myocardial energetics exploiting the specific relaxation properties of different molecules add promising and radiation-free strings to the bow of the modality. Given the unmatched amount of information that can be obtained from a single scan, with increasingly faster and versatile protocols, CMR imaging will certainly add in the following years meaningful pieces to this complex puzzle.

## Data Availability

No datasets were generated or analysed during the current study.
